# Supplementary figures and images for: Combining Low-Dose Computer-Tomography-Based Radiomics and Serum Metabolomics for Diagnosis of Malignant Nodules in Participants of Lung Cancer Screening Studies
Source: Biomolecules. 2023 Dec 28;14(1):44. doi: 10.3390/biom14010044 (PMC10813699; doi:10.3390/biom14010044)

**A)**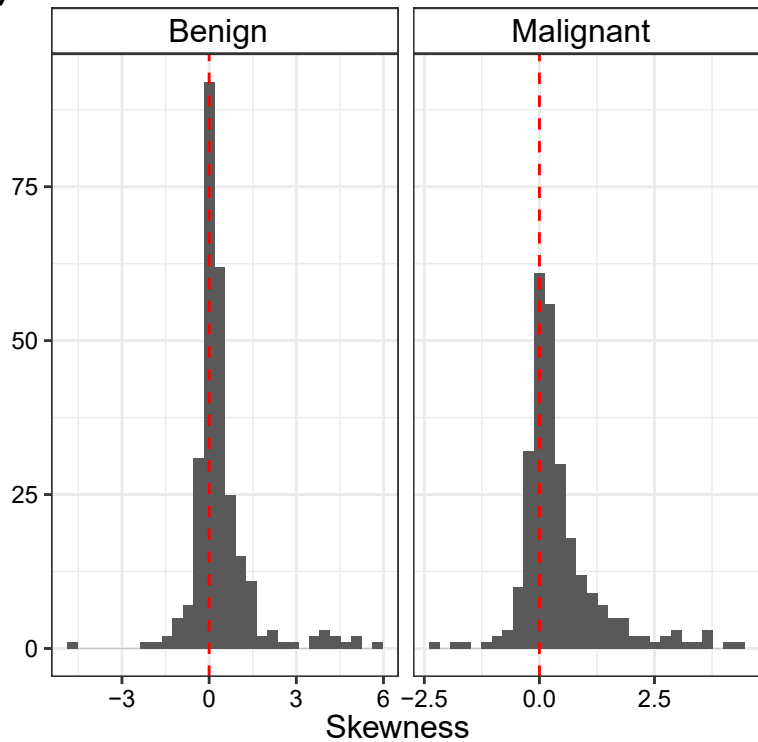**B)**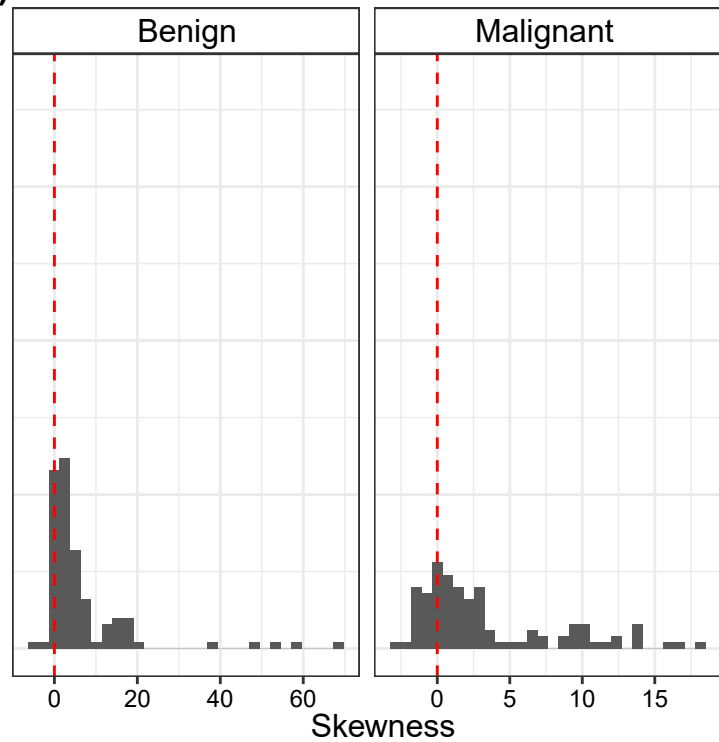

Supplement: Supplementary file 1 [file biomolecules-14-00044-s001.zip › Supp_files/FigS2.pdf]

**A)**

## Metabolomics

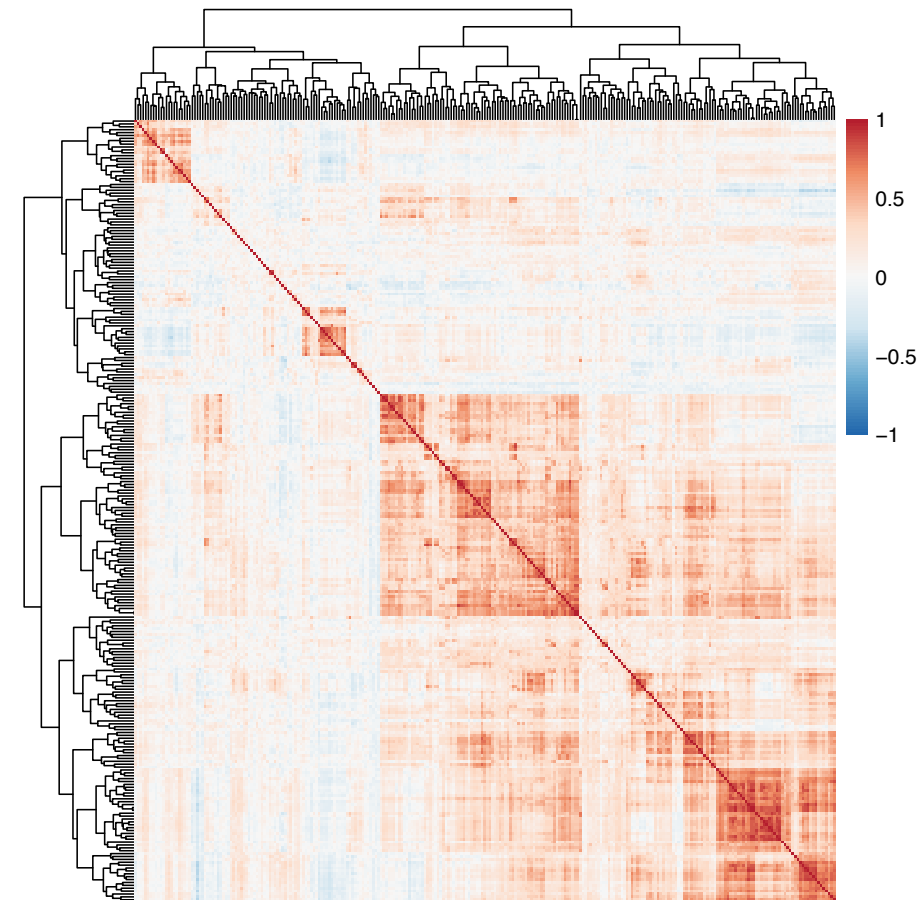**B)**

## Radiomics

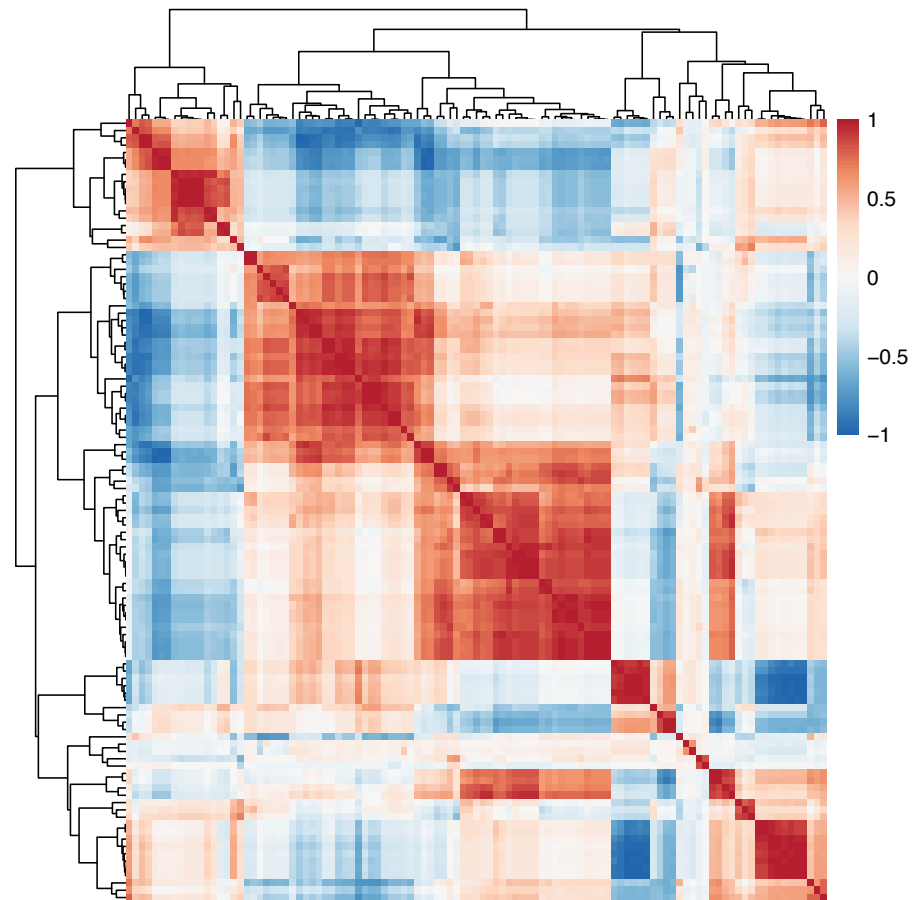

Supplement: Supplementary file 1 [file biomolecules-14-00044-s001.zip › Supp_files/FigS3_Corr_features.pdf]

A)

## Metabolomics

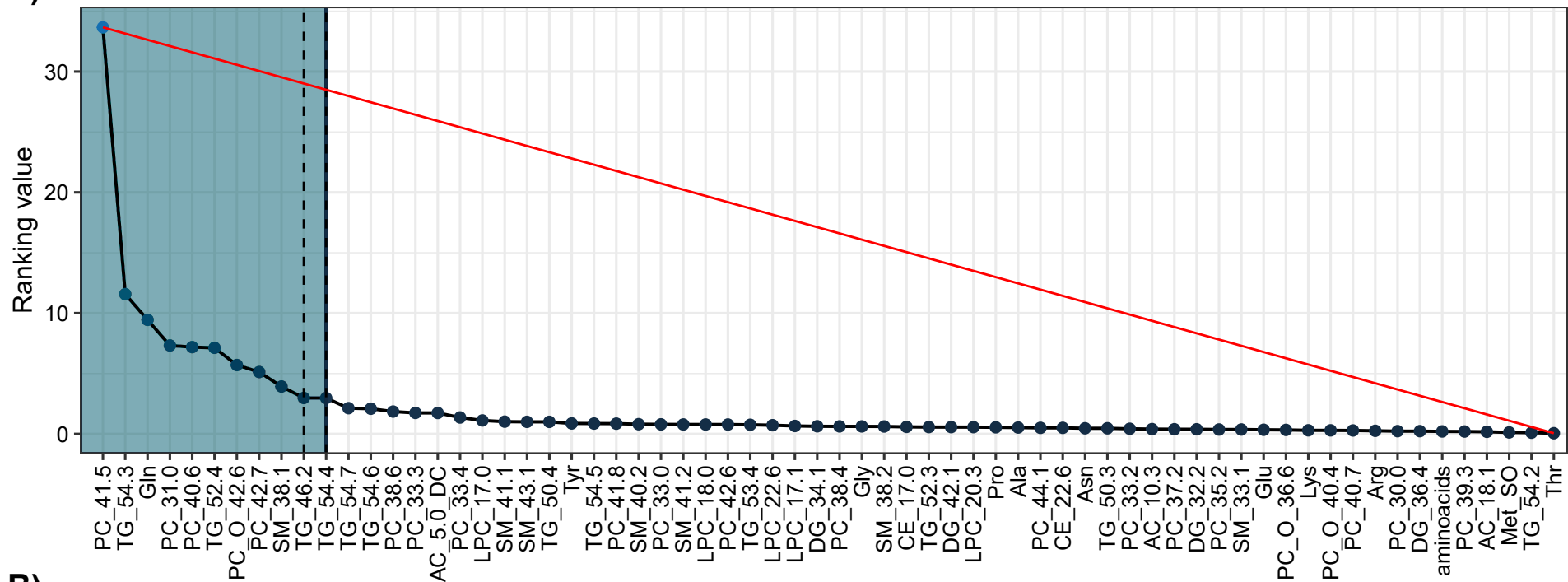

B)

## Radiomics

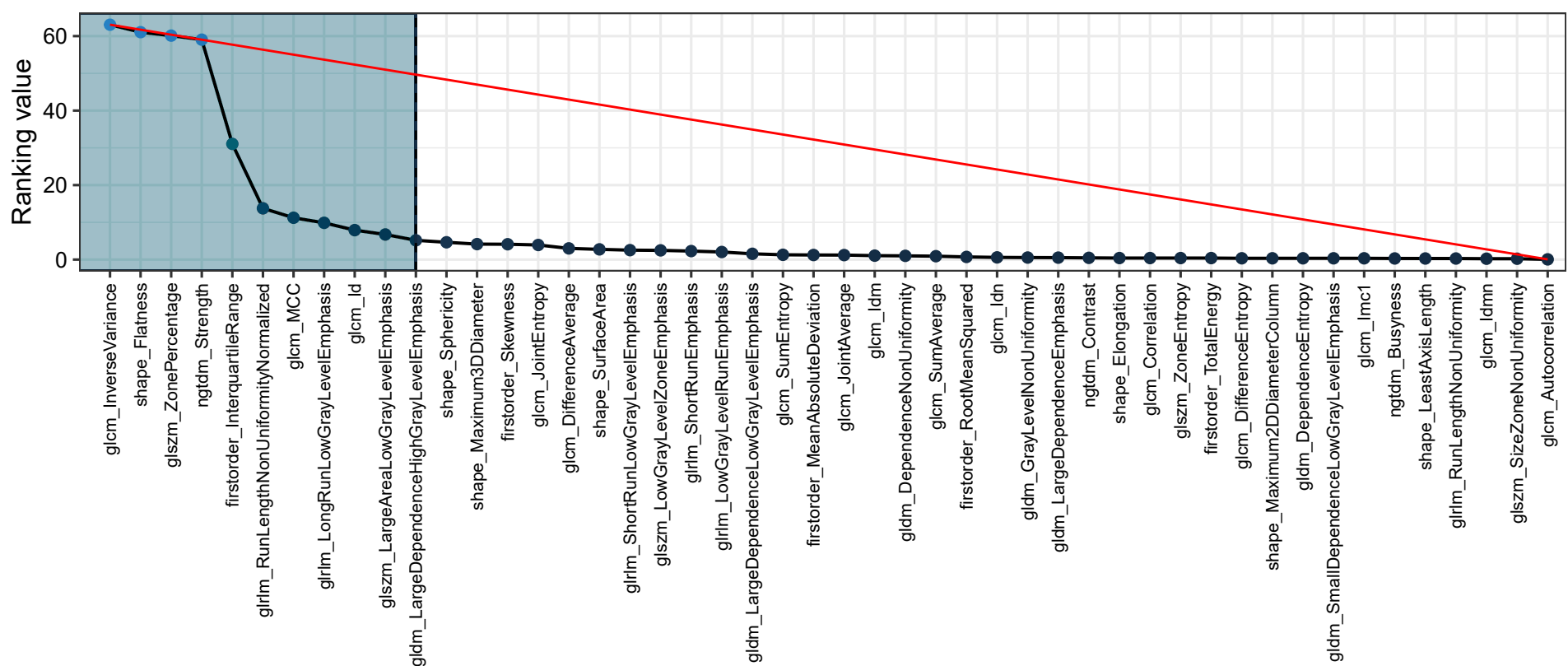

Supplement: Supplementary file 1 [file biomolecules-14-00044-s001.zip › Supp_files/FigS4_LR.pdf]

A)

## Metabolomics

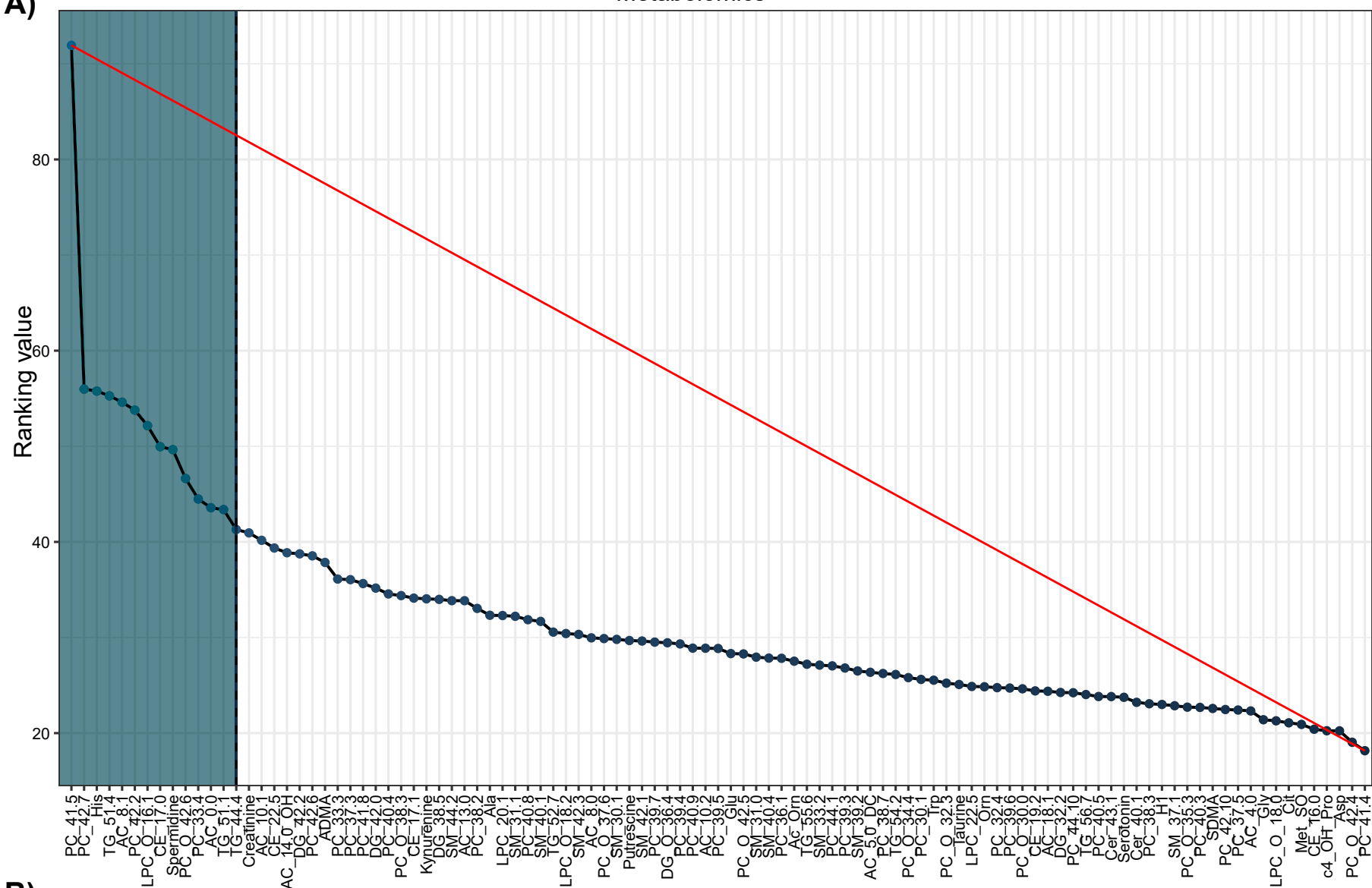

B)

## Radiomics

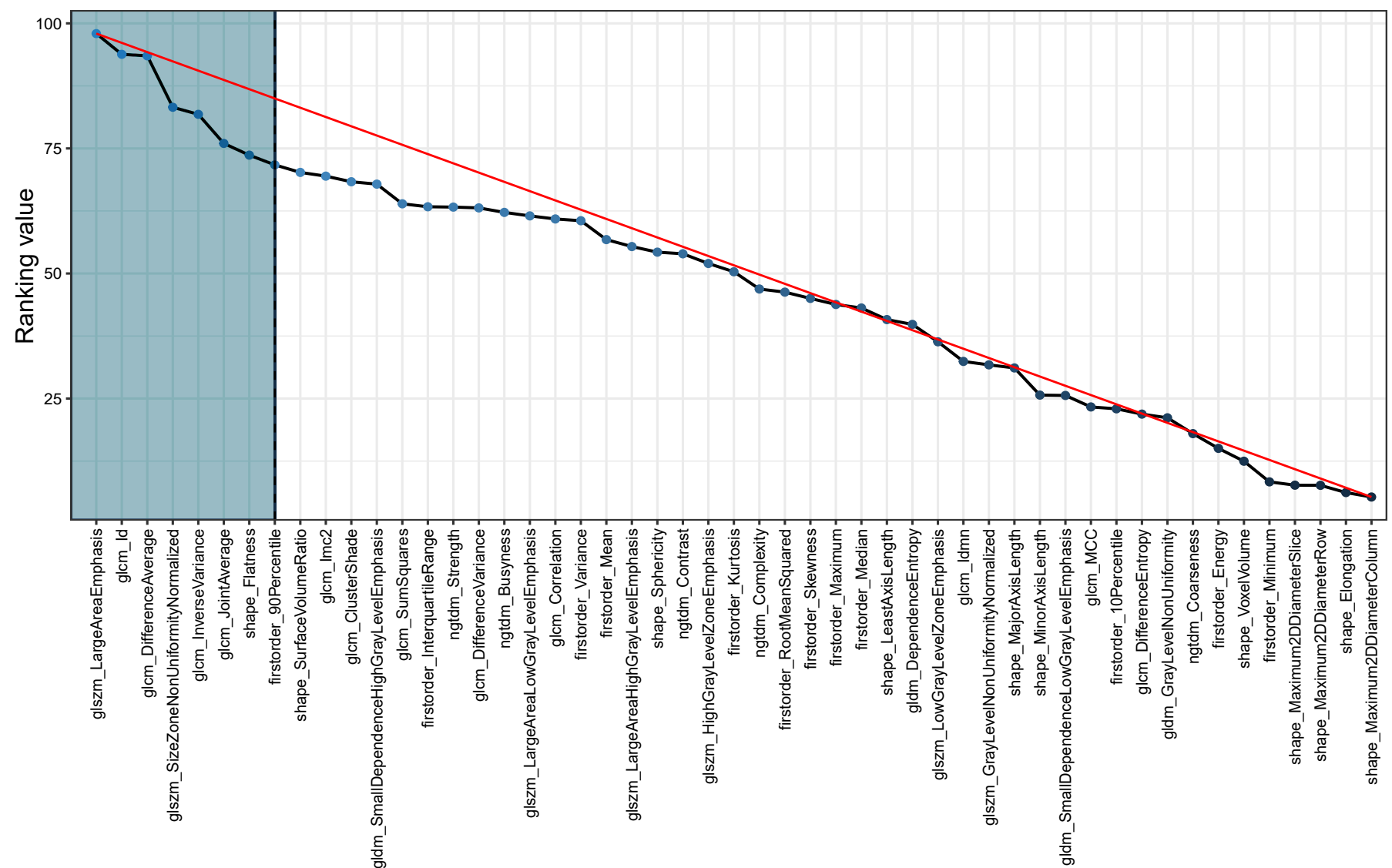

Supplement: Supplementary file 1 [file biomolecules-14-00044-s001.zip › Supp_files/FigS5_RF.pdf]

**A)**

## Metabolomics

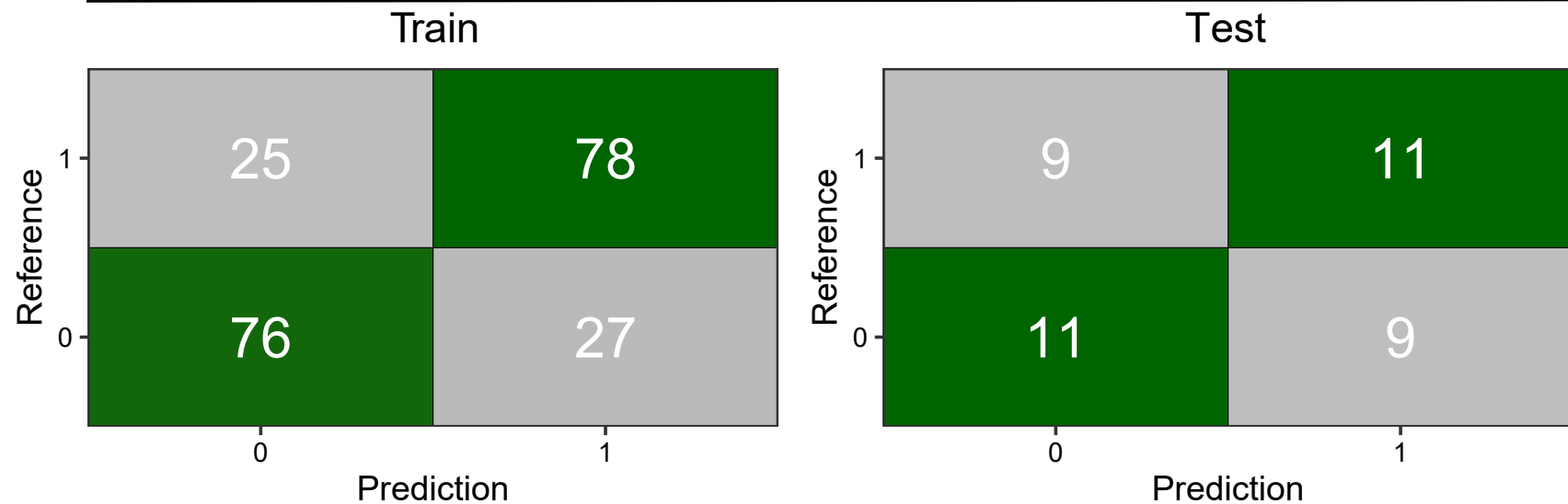**B)**

## Radiomics

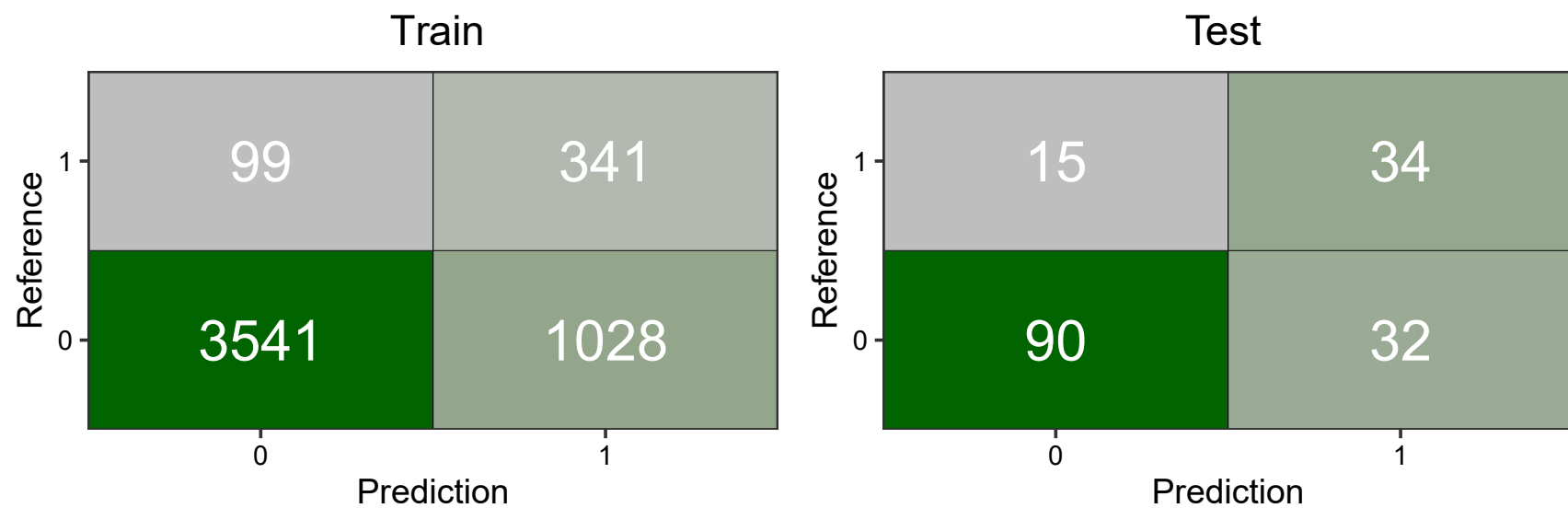

Supplement: Supplementary file 1 [file biomolecules-14-00044-s001.zip › Supp_files/FigS6_CM_heat_LR.pdf]

**A)**

Metabolomics

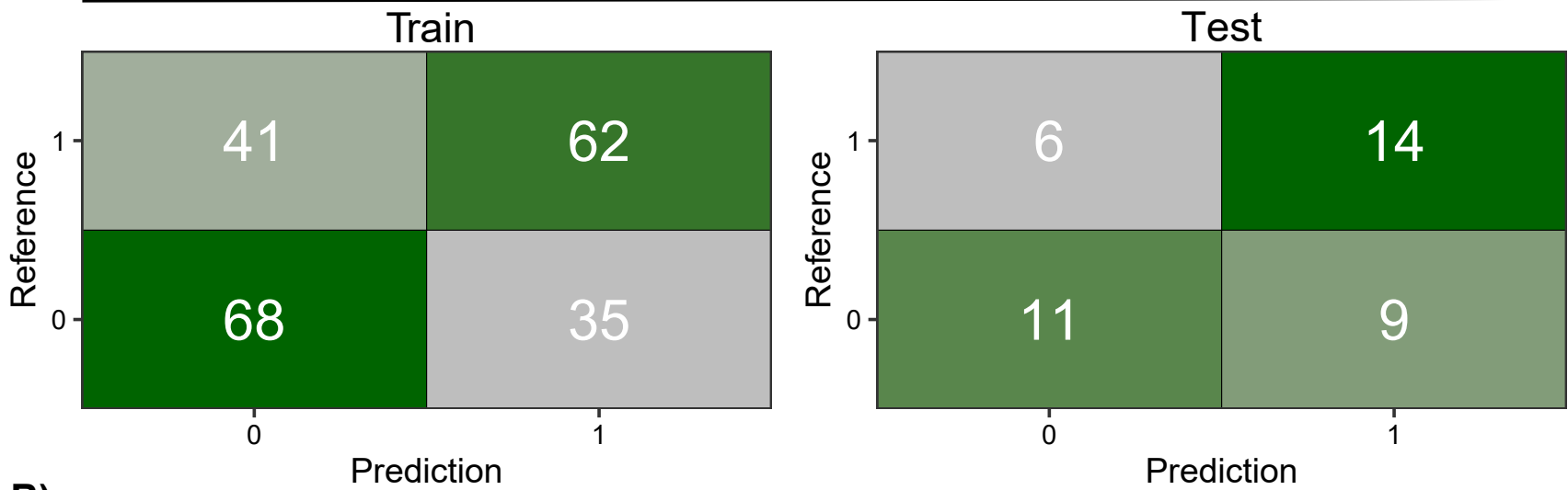

**B)**

Radiomics

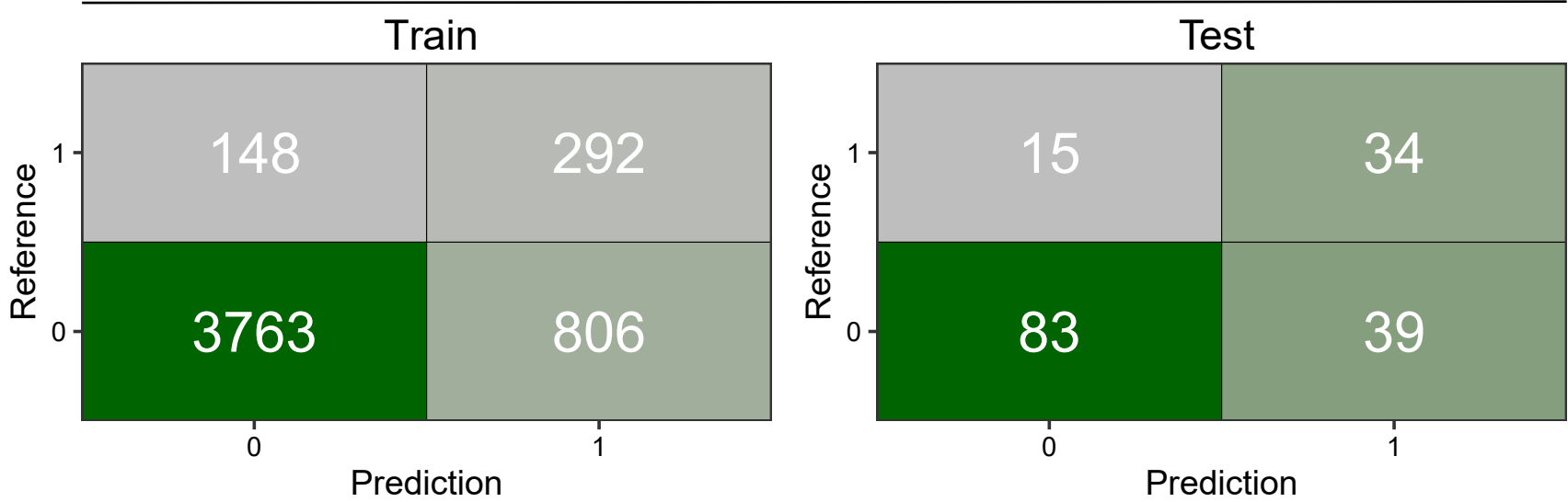

Supplement: Supplementary file 1 [file biomolecules-14-00044-s001.zip › Supp_files/FigS7_CM_heat_RF.pdf]

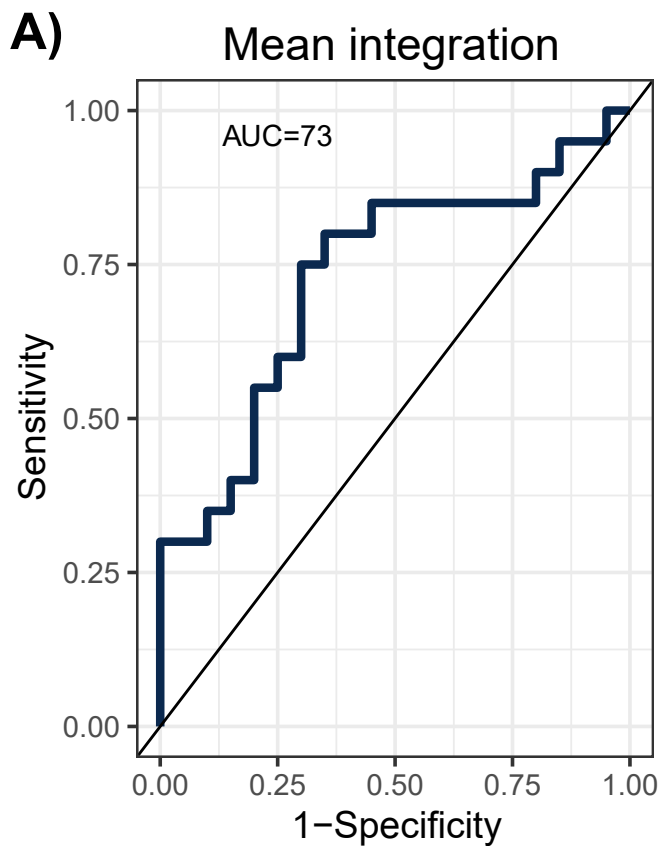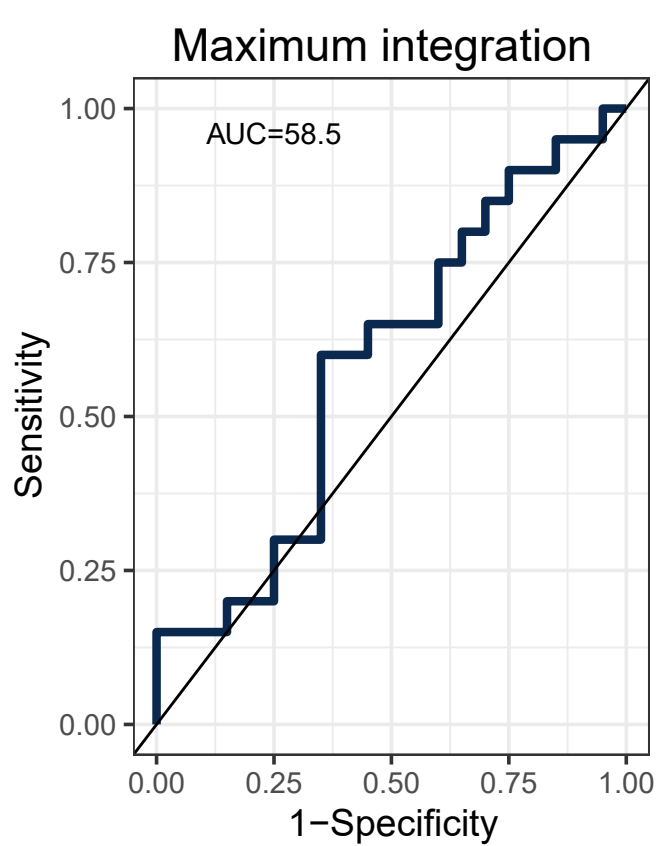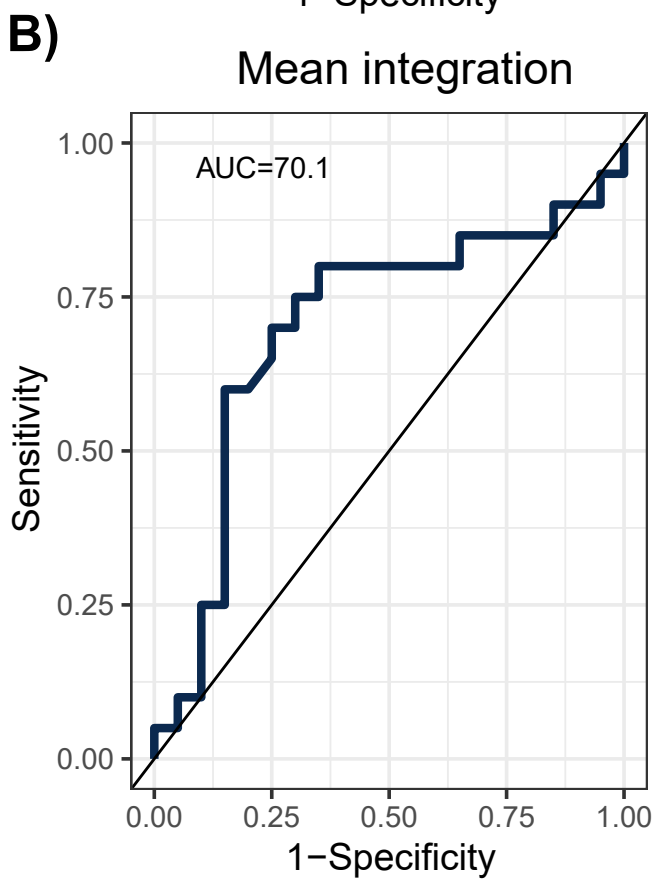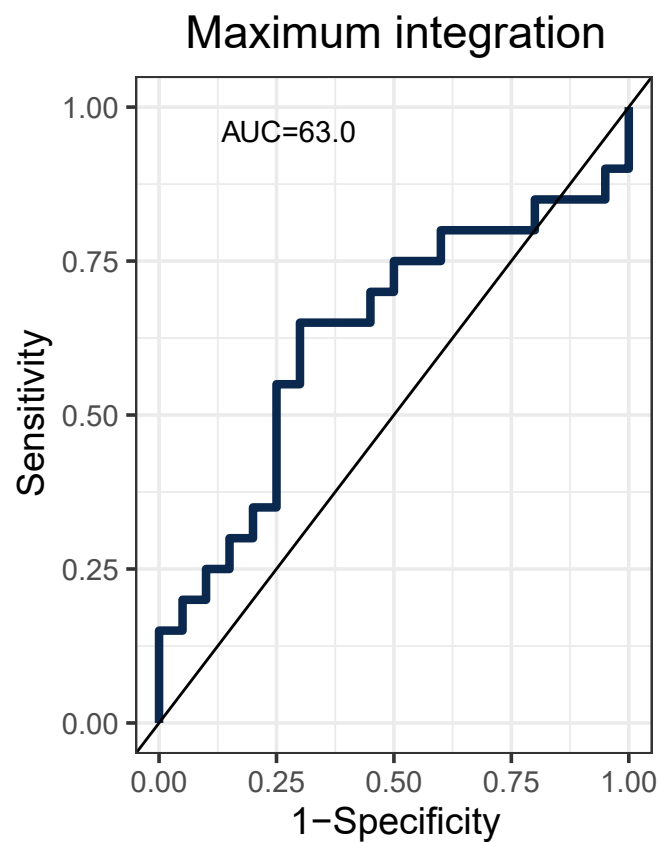

Supplement: Supplementary file 1 [file biomolecules-14-00044-s001.zip › Supp_files/FigS8_other_integration.pdf]

**A)****Metabolomics**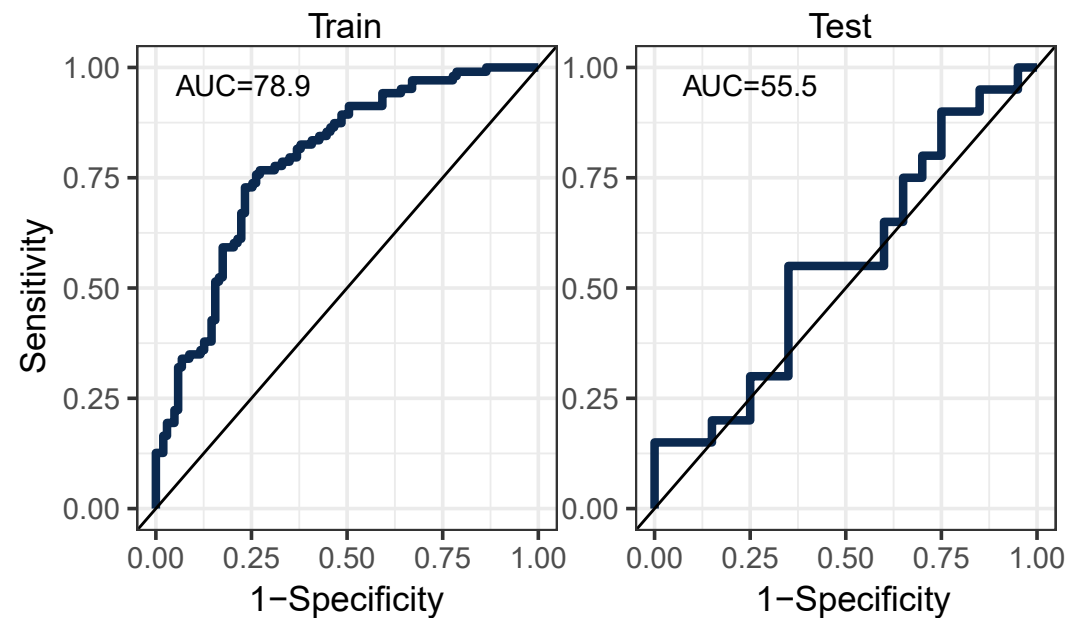**Radiomics**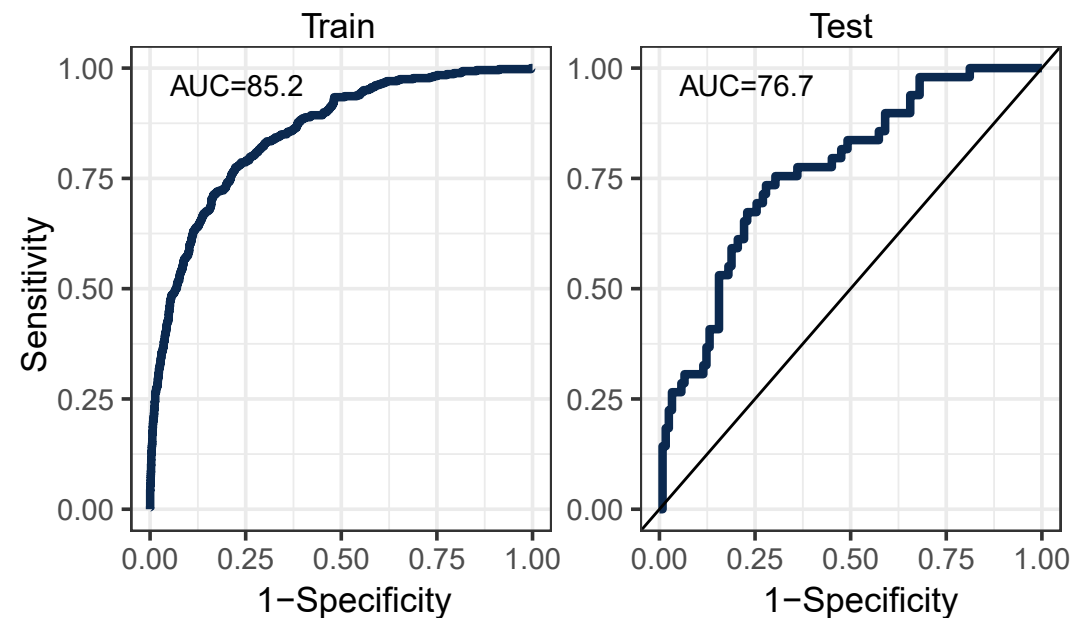**B)****Metabolomics**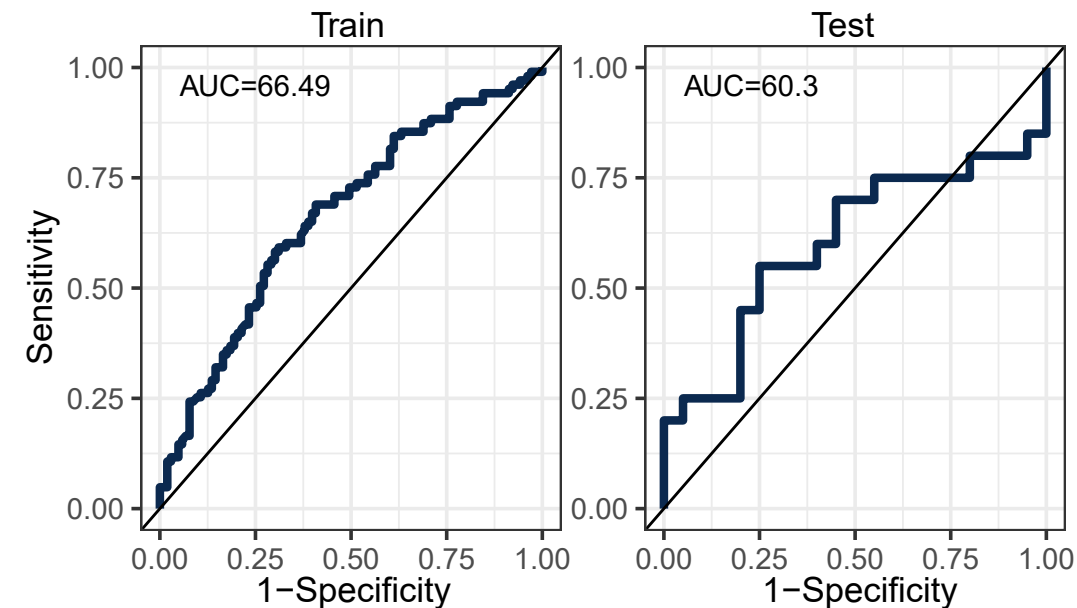**Radiomics**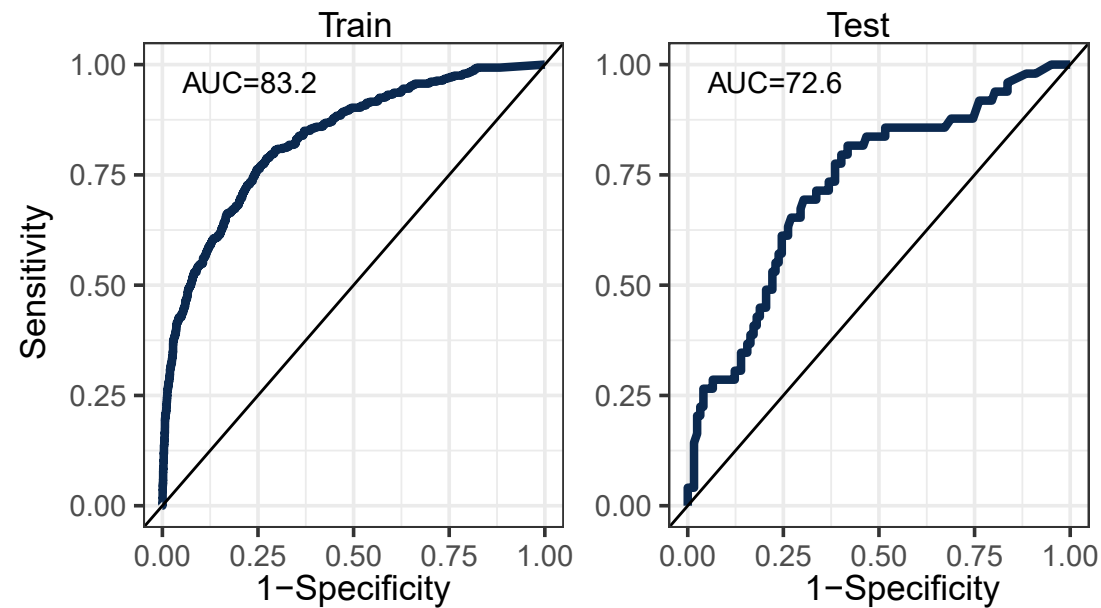

Supplement: Supplementary file 1 [file biomolecules-14-00044-s001.zip › Supp_files/FigS9_AUC_train_test.pdf]
