# Supplementary material for: Combining Low-Dose Computer-Tomography-Based Radiomics and Serum Metabolomics for Diagnosis of Malignant Nodules in Participants of Lung Cancer Screening Studies
Source: Biomolecules. 2023 Dec 28;14(1):44. doi: 10.3390/biom14010044 (PMC10813699; doi:10.3390/biom14010044)
Supplement: Supplementary file 1 [file FigS1.pdf › Supp_files/FigS1.pdf]

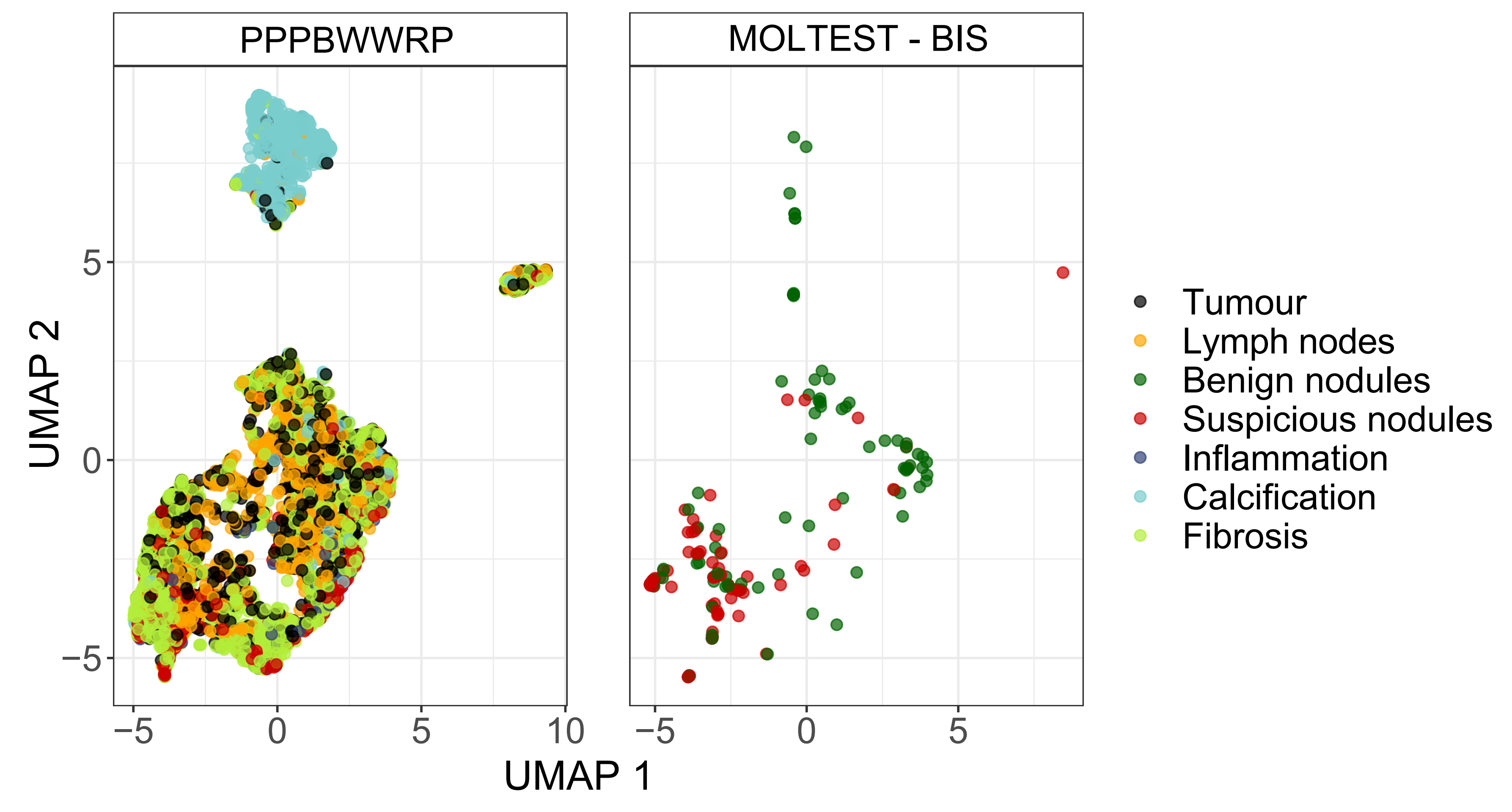
PPPBWWRP
MOLTEST - BIS
UMAP 2
UMAP 1
5
0
−5
−5
0
5
10
−5
0
5
Tumour
Lymph nodes
Benign nodules
Suspicious nodules
Inflammation
Calcification
Fibrosis
